# Evaluation of adherence to anti-rabies vaccination schedule and its predictive factors at Addis Alem hospital, Bahir Dar, Ethiopia

**DOI:** 10.1038/s41598-025-13320-9

**Published:** 2025-08-28

**Authors:** Ebrahim Abdela Siraj, Tadele Behulu, Sosina Shumye, Wondmalem Gebral, Beselam Gizachew, Adugna Tasew Tebabal, Ashagrachew Tewabe Yayehrad, Selamawit Yimer Kebede, Gizachew Motbaynor, Zenaw Debasu Addisu

**Affiliations:** 1 Department of Pharmaceutics, School of Pharmacy, College of Medicine and Health Science, Bahir Dar University, Bahir Dar, Ethiopia; 2 GAMBY Medical and Business College, Bahir Dar, Ethiopia; 3 Department of Pharmacognosy, School of Pharmacy, College of Medicine and Health Science, Bahir Dar University, Bahir Dar, Ethiopia; 4 Department of Medicinal Chemistry, School of Pharmacy, College of Medicine and Health Science, Bahir Dar University, Bahir Dar, Ethiopia; 5 Department of Clinical Pharmacy, School of Pharmacy, College of Medicine and Health Science, Bahir Dar University, Bahir Dar, Ethiopia

**Keywords:** Rabies, Post-exposure prophylaxis, Vaccination adherence, Ethiopia, Anti-rabies vaccine, Immunology, Microbiology

## Abstract

Rabies is a dangerous viral neglected tropical disease and infects humans, causing big problems for health authorities in Ethiopia. Though PEP is available, still there is insufficient awareness, difficulties of accessing to healthcare and logistics issues still make it hard for some to properly follow the rabies vaccination schedule. The primary aim of this study is to measure how properly the anti-rabies vaccine is given and to determine which factors influence the schedule among patients in Addis Alem General Hospital, Bahir Dar.From June to July 2024, a facility-based cross-sectional study was set up with 190 participants who were initiating rabies vaccination. Data were collected by using planned questionnaires and reviewing charts. To study both adherence rates and their causes, we used descriptive statistics and multivariate logistic regression and considered results significant if *p* < 0.05. Adherence rates declined significantly across vaccination doses that could be due to several factors such as vaccine hesitancy, misinformation, lack of access to follow-up doses, or diminished perceived risk after the initial dose. While each participant has received the first shot, but fewer received the second and those numbers dropped further for the third and fourth, fifth doses: 97.3%, 95.7% and 94.7%, 93.6% respectively. The majority or 81.6%, displayed good adherence. Significant factors predicting better adherence were being aged 20–40 years (2.15 times the odds, *p* = 0.023), having only basic education (2 times the odds, *p* = 0.027) and residing a short distance (5 km or less) from a healthcare facility (2.49 times the odds, *p* = 0.042). Concerningly, over 40% of those surveyed recognized that they should have started PEP at least 4 days ago but did not and only 39% knew that an anti-rabies vaccine was available prior to this. The findings highlight critical gaps in knowledge and timely access to rabies PEP, despite relatively high initial vaccine uptake. Targeted interventions such as public education, decentralized vaccine distribution, and cost-reduction strategies are essential to improving adherence and achieving the WHO’s 2030 rabies elimination goal.

## Introduction

Rabies is one of the neglected tropical diseases that primarily affects animals and also poses a threat to humans worldwide. Infected animals often transmit the disease through bites or scratches. Due to its dual burden on the developing world, the World Health Organization has classified rabies as one of the 20 listed NTDs, with a focus on elimination ^1,2^. It remains a significant public health concern, particularly in developing countries, and is underreported compared to other health conditions. Globally, rabies causes nearly 59,000 deaths annually, with the majority of cases reported from Asia and the African continent. It is estimated that around 55,000 people die from rabies each year, with approximately 44% of these deaths occurring in African countries ^3^. Specifically, in Ethiopia, over 10,000 people die from rabies annually, highlighting the severity of this public health burden and emphasizing the need for comprehensive and well-organized interventions for prevention and treatment ^4^.

Universally, dogs and other animals living closely with humans are the main sources of rabies transmission to people. The common signs and symptoms of rabies include fever, mood changes, fear, difficulty swallowing water (hydrophobia), sensitivity to light (photophobia), involuntary and excessive salivation, and ultimately death if not treated early ^5^. Therefore, primary preventive measures such as post-exposure prophylaxis (PEP) should be administered before the incubation period ends after a known exposure. PEP involves thorough wound cleaning, administration of rabies immunoglobulin, and a series of vaccinations given over consecutive days ^6^. Despite the fact that rabies is a preventable disease, it remains a major public health challenge in underdeveloped countries due to low awareness, inadequate access to vaccines, and limited healthcare facilities ^7^. In some countries with strict dog control and vaccination programs, rabies has been eliminated from domestic dog populations. However, in developing nations, including Ethiopia, rabies continues to be a problem due to the presence of many stray dogs, poor vaccination coverage, and inadequate surveillance programs ^8,9^. Furthermore, as the disease affects a large number of people, public health measures such as mass vaccination and awareness campaigns play a crucial role in preventing the spread of the disease ^10^.

Even though rabies has posed challenges for a long time, there is still insufficient data regarding the number of people affected, vaccine coverage, and other relevant information. Human rabies in Ethiopia is mainly caused by dog bites and results in many deaths ^11^. Although it is known that rabies can be prevented with a predetermined anti-rabies vaccination schedule, poor access to vaccination programs, affordability issues, limited public knowledge, and the inability to strictly follow recommendations contribute to continued cases ^3^. In the Ethiopian context, the Ministry of Health and the Ministry of Agriculture, in collaboration with the Ethiopian Public Health Institute, conduct mass vaccination campaigns for both humans and animals. Other stakeholders and international organizations such as WHO, FAO, GARC, and USAID provide technical and financial support to aid vaccination and advocacy programs. China integrates various sectors to work together toward a common goal, including public health and animal health institutions along with environmental protection agencies. Despite many vaccines being provided during World Rabies Day, vaccination coverage and access to PEP remain challenges. For instance, Ethiopia has developed a National Rabies Control and Elimination Strategy that runs from 2018 to 2030, aiming to eliminate rabies by 2030 through a One Health approach. Various preventive measures include annual dog vaccination, improving post-exposure responses, and developing a platform to enhance public awareness and implement stricter control of rabies. Joint programs operating under a nationwide One Health approach involve a multidisciplinary committee composed of members from the Ministry of Health and the Ministry of Agriculture, in collaboration with WHO and FAO ^12^.

Despite challenges in vaccination access and public awareness, Ethiopia has shown progressive changes through the collaborative efforts of various stakeholders, training programs, and foreign aid. According to global studies, enhancing public awareness, implementing mass vaccination programs, and increasing the availability of PEP are significantly important steps for the effective control and prevention of rabies. Similar findings from Tanzania, India, and the Philippines indicated that poor knowledge and inadequate vaccine supply pose major challenges to rabies control ^13^. However, studies conducted at the community level are still insufficient, possibly due to ethical concerns. It is estimated that nearly 230,000 to 300,000 dogs are present in the country, with the vast majority living on the streets. The burden is further exacerbated by the fact that these dogs do not have owners to care for them, making it difficult for the government to vaccinate them—thus contributing to the spread of rabies. Between the years 2013 and 2019, over 3,100 cases of dog bites were reported from various sites across the country ^14^.

The main rabies prevention efforts carried out by EPHI in Ethiopia include providing five vaccine shots and rabies immunoglobulin to individuals who were not previously vaccinated and are exposed to the disease through bites or scratches. The vaccine is locally prepared and administered by veterinary experts to animals, mainly dogs, in accordance with the Ministry of Agriculture’s guidelines. In recent years, free mass dog vaccination campaigns have been conducted, although the exact figures are not adequately documented. Community education programs are also implemented through various campaigns, though concerns have been raised about their consistency and outcome-based learning with measurable results ^15,16^. However, the study on covid vaccination acceptance showed that vaccination reception rates declined significantly across vaccination doses due to various factors such as vaccine hesitancy, misinformation, lack of access to follow-up doses, or diminished perceived risk after the initial dose ^17^. In Ethiopia, vaccines are provided both at the household level and through national supply channels. The Ethiopian government rely on WHO advice for rabies control, recommend five-dose vaccination after a bite and give rabies vaccines to risky groups before an exposure. The emergency department and Pharmacy units administer (vaccines) which usually amount to about five doses, although there are at times shortages. The vaccine is available though there is frequent stockouts and often health education is offered to clients. Usually, the government increases awareness about rabies among people and involves many fields in One Health programs ^15,16^.

Hence, to address the aforementioned gaps, this study emphasizes adherence to anti-rabies vaccination schedules and the predictive factors that could affect compliance at the anti-rabies clinic of Addis Alem Hospital. This study will help identify key factors to consider and assist in designing appropriate strategies to increase vaccination rates, prevent the spread of the disease, and save human lives. Therefore, public health experts, scientists, and other stakeholders may obtain useful information from this study to develop customized strategies for controlling rabies in Ethiopia and other countries with similar conditions.

## Method

### Study area and period

Our study was performed at Addis General Hospital (AGH) in Bahir Dar, the capital of the Amhara region in Northwest Ethiopia. Bahir Dar lies around 560 km away from Addis Ababa. Within its many departments and wards, AGH includes the outpatient department (OPD), medical, gynecology and obstetrics, pediatric and surgical units. In addition, there is care for emergencies, maternal and children’s health, a psychiatry clinic, laboratory services, X-ray facilities and aftercare for chronic diseases such as TB, DM and HIV/AIDS. The hospital has outpatient and inpatient pharmacies. The research was carried out in the medical ward of AGH between June 1 and July 1, 2024.

### Study population

During the data collection period, our research involved anyone who visited the AGH rabies clinic after receiving the anti-rabies vaccine. All rabies vaccine users who attended follow-ups at the AGH clinic were included in the source population. Individuals included in the study were at the clinic at the appropriate time, got the rabies vaccine and were able as well as willing to answer the questionnaire. Anyone who did not help voluntarily, was sick and didn’t respond or whose interview was cut off was excluded from this study.

### The study design

The study used a cross-sectional approach by letting the outcome variable represent whether a patient got the full 5 dose vaccination schedule. A review of previous charts was carried out during one month to determine if patients had completed their vaccination series. Patients whose fifth dose was given during the review period were marked complete on their chart and those who had fewer doses were checked to see if they received the fifth dose.

### Eligibility criteria and sample size determination

To be eligible, a person had to be willing, able to answer and present throughout the study, but those who refused or could not take part in the interviews were not included.

**Fig. 1:**
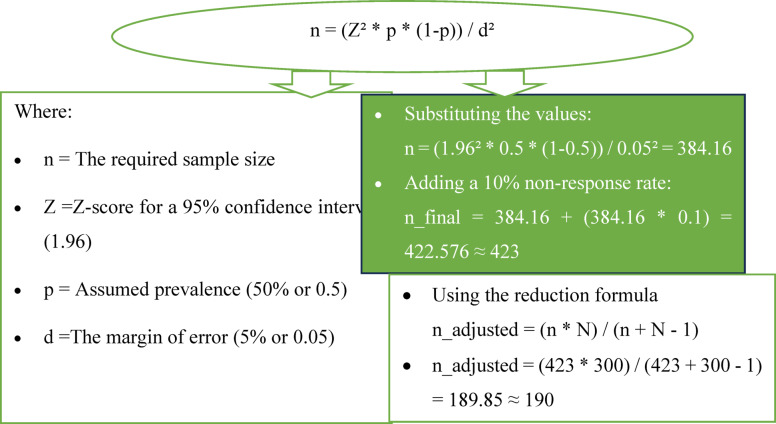
Sampling size calculation.

Using a questionnaire, we established how many people had been vaccinated against rabies and the independent variables in our study were age, gender, religion, ethnicity, parental education level, economic status and occupation. The sample size was first computed using the single population proportion method (Fig. 1).

Therefore, the research included a total of 190 participants. The sample size was reached by using a method where suitable participants were recruited. Data were obtained by talking with respondents and reviewing their charts to confirm if their vaccination course was complete by the period of the study.

### Operational definition

#### Good adherence

A patient should receive every required dose of the anti-rabies vaccine within the allowed grace period (1–2 days) for each day (according to the standard schedule, Day 0, 3, 7, 14 and 28).

#### Poor adherence

A patient displays poor adherence when they miss one or more vaccination doses entirely, take a shot late by more than two days or quit vaccination partway through the schedule.

### Sampling technique and data collection

Participants were enrolled one after another until the study team had gathered 190 submissions. Since all the observations were made at the clinic, researchers decided that this approach would be the most convenient. Responses were gathered by interviewing participants in person, using a questionnaire. The tool aimed to gather needed sociodemographic information and details about the use and keeping of rabies vaccines.

### Data collection procedure, quality management and analysis

A questionnaire and chart review designed from similar studies in the region and adjusted for proper use, were used to collect data. Three data collectors were trained well ahead of the data collection period. A pretest was carried out at another place with a sample of 5% to see if the questionnaire was precise and reliable and to make appropriate improvements and necessary modifications was made in considering the feedbacks in the pretest before the actual data collection. The pretest showed a Cronbach’s alpha of more than 0.8, suggesting that the tool can be trusted and used on the main medical charts.

The collected data were initially checked if they had no errors and were complete before moving them to SPSS version 26.0 for analysis. Incomplete, errors data were discarded and not included in the study. The Findings were first summarized using descriptive statistics and both bivariate and multivariate methods were used to look for predictors. For bivariate analysis, only variables whose significance level was *p* ≤ 0.25 were chosen for the multivariate model. We considered variables to be statistically important if their adjusted odds ratio was below 0.05 and their 95% CI did not include 1.

## Results

### Sociodemographic characteristics

The clients’ adherence to immunization against rabies and what affected this at Addis Alem General Hospital’s rabies clinic in Northwest Ethiopia. The study included 190 people and there were more males (55.3%) than females (44.7%). More than two out of three (66.3%) were living in rural areas, as compared to those (33.7%) living in urban areas. Most participants were under 20 (53.7%), then 20 to 40 years old (38.4%) and finally over 40 years old (7.9%). Among the population, 50% had only finished primary school, 24.7% were educated to high school level and the rest, 25.3%, could not read or write. Of the students, nearly half (40.5%) were involved, followed by farmers (23.2%), housewives (15.3%), merchants (8.4%) and other types. Almost two-thirds of the participants were single, while 33% of participants said they were married (Table 1).

**Table 1:** Socio-demographic characteristics of the study participants received treatment ARV clinic at Adiss Alem General Hospital, northwest Ethiopia, 2024 (n = 190)*.*

Variable	Category	Frequency	Percentage (%)
Age	< 20	103	54.2
	20–40	50	26.3
	> 40	37	19.5
Sex	Male	135	71.1
	Female	55	28.9
Residence	Rural	139	73.2
	Urban	51	26.8
Educational status	Illiterate	105	55.3
	Primary	36	18.9
	Secondary	27	14.2
	College and above	22	11.6
Occupation	Governmental employee	49	25.8
	Private employee	36	20.5
	Merchant	26	13.7
	Housewife	28	14.7
	Student	5	2.6
	Farmer	43	22.6
Marital status	Married	137	72.1
	Single	29	15.3
	Widowed	13	6.8
	Divorced	11	5.8
Religious	Orthodox	124	61.1
	Muslim	50	26.3
	Catholic	8	4.2
	Protestant	11	5.8
	Others	5	2.6
Monthly income	< 50 USD	19	10
	50–100 USD	39	20.5
	150–200 USD	41	21.6
	> 200 USD	91	47.9

### Assessment to adherence and predicting factors

**Fig. 2:**
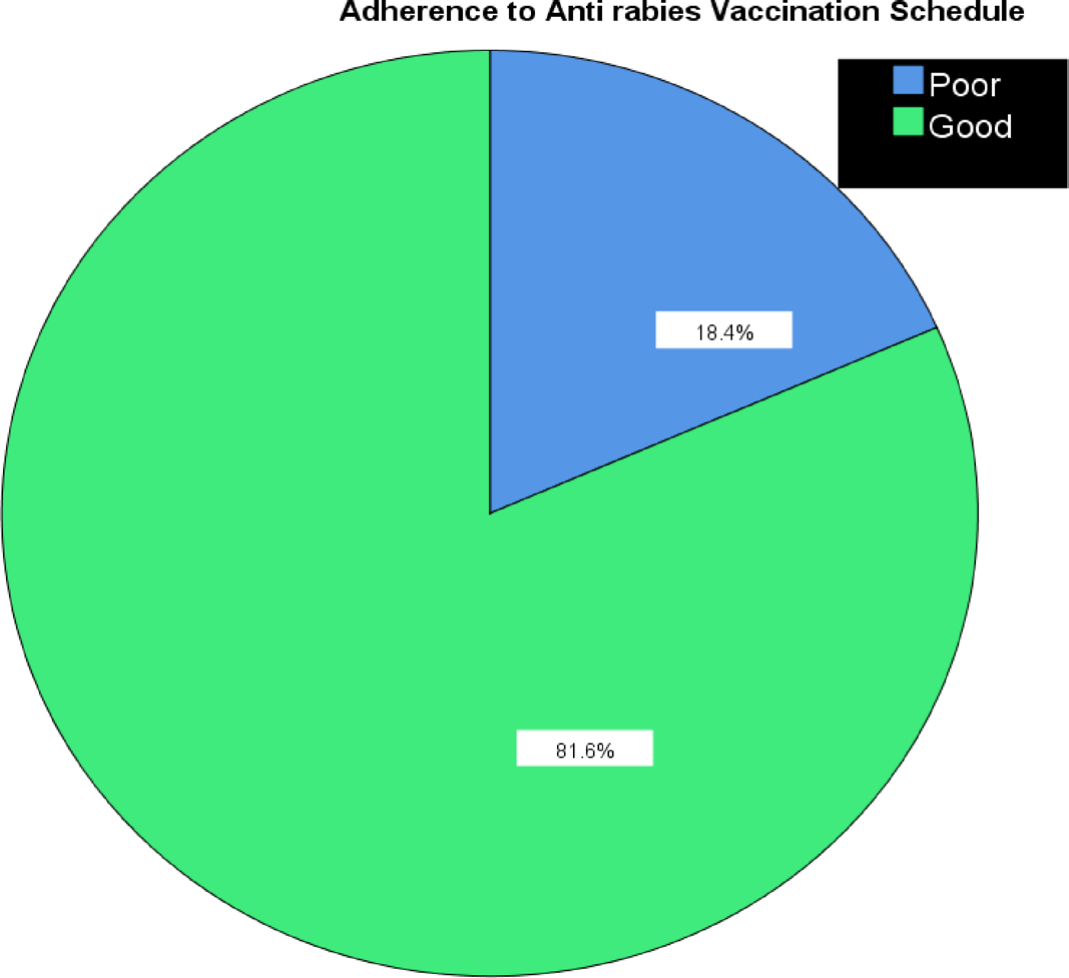
Adherence to Anti rabies Vaccination Schedule.

There was considerable range in how well the anti-rabies vaccine schedule was kept based on the dose. All participants had the first dose, but only 85.3% received the second, 37.9% had the third and 18.9% had the fourth dose. Most participants were given the vaccine through a shallow injection into the upper skin layer (82.6%) and some with a deeper shot in the lower skin (17.4%). It took some time for many people to begin post-exposure prophylaxis (PEP). Only 1 in every 7 people had the shot on the same day as their animal bite, but more people (about 2 in 4) waited between three days and a week. Alarmingly, 10.5% did not reach medical care until more than one week after contact with a possibly infected animal, so they were significantly more likely to get rabies. Of the participants, 72.6% knew something about rabies, but 27.4% had not heard about the disease before. Almost two-thirds (61.1%) of participants said they were unaware and only 38.9% could answer correctly about anti-rabies vaccination. A lot of the information was gathered via radio (35.1%), from family (28.4%), television (23%), school (17.6%) and newspapers (6.8%). A major reason for bites in America were dogs (92.6%), followed by cats (5.8%) and wild animals (1.6%). Because many rabies cases involve dogs, efforts to hold stray dogs and encourage responsible pet care are needed in the fight against rabies (Table 2). The majority (81.6%) of participants followed the vaccination schedule well and about 18.4% did not strictly follow the predetermined vaccination schedule. The results reveal that the population mostly followed the rules (Fig. 2).

**Table 2:** Assessment of adherence to anti-rabies vaccination schedule and associated factors among the clients in the rabies clinic at Addis Alem General Hospital, Bahir Dar, Ethiopia.

Characteristics	Category	Frequency	Percentage (%)
Ever heard of the Rabies virus previous	Yes	138	72.6
	No	52	27.4
Knowledge about rabies symptoms	Yes	73	38.4
	No	117	61.6
Knowledge about anti-rabies vaccination	Yes	106	55.8
	No	84	44.2
Source of information about Anti-Rabies Vaccination in the previous	Radio	69	36.3
	Newspaper	43	22.6
	Family	68	35.8
	TV	4	2.1
	School	6	3.2
Distance from the health facility	< 5 km	21	11.1
	5–20 km	108	56.8
	> 20 km	61	32.1
Access to health facility	Difficult	78	41.1
	Neutral	73	38.4
	Easy	39	20.5
Animals bite	Dog	151	79.5
	Cat	27	14.2
	Wild	12	6.3
Knowledge about susceptibility to host	Yes	138	72.6
	No	52	27.4
ARV vaccine received	1 st dose	190	100
	2 st dose	185	97.3
	3 st dose	182	95.7
	4th dose	180	94.7
	5th dose	178	93.6
Route of administration	Cutaneous	147	77.4
	Subcutaneous	43	22.6
Missed dose	Yes	35	18.4
	No	155	81.6
Time gap between animal bite and first dose post-exposure vaccine intake	0 day	26	13.7
	1–3 days	63	33.2
	4–7 days	81	42.6
	> 7 days	20	10.5
What do you think of completing the rabies vaccination schedule	Not Important	36	18.9
	Neutral	55	28.9
	Important	99	52.1

Those between 20 and 40 years of age were more likely to follow the vaccination plan than those younger than 20 years (AOR = 2.15, *p* = 0.023). They are more health aware or can move around more freely than before. Being educated up to primary level was linked with greater odds of adherence, but having no schooling was not (AOR = 2.0, *p* = 0.027). Being educated probably helped parents become more aware of how important it is to follow the vaccine calendar. Those who live less than 5 km away from the health center follow the treatment plan better than those who live more than 20 km away (AOR = 2.49, *p* = 0.042). In three regions, having accessible medical facilities helped more residents receive the vaccine on time. We did not find evidence of a statistically significant link between the outcomes and sex, occupation or income after considering their effects together (Table 3).

**Table 3:** Factors associated with the adherence of anti-rabies vaccination among victims in the Adis Alem general hospital from Jun to July 30, 2024.

Predictor variables		Adherence to anti rabies vaccination		COR 95% CI	*P* value	AOR 95% CI	*P* value
		Poor	Good				
Age	< 20	20	82	0.97 (2.96–14.55)	0.0318	1.77 (0.02–1.31)	0.090
	20–40	13	60	0.86 (1.55–6.84)	0.072*	2.15 (0.030–0.78)	0.023*
	> 40	3	12	1		1	1
Sex	Male	24	81	1.8 (1.35–7.58)	0.230*	2 (1.63–6.57)	0.752
	Female	12	73	1	1	1	1
Religious	Orthodox	22	71	2.0 (4.16–8.21)	0.420	5.30 (0.17–1.68)	0.278
	Muslim	12	60	1.3 (0.78–7.97)	0.230*	2.41 (0.002–0.99)	0.069
	Catholic						
	Protestant						
	Others	2	13	2.13 (2.23–8-58)	0.581	1.61 (0.51–3.14)	0.760
Educational status	Illiterate	8	40	4.50	0.004*	3.52 (0.62–12.21)	0.092
	Primary (1–8)	22	73	1.36 (1.23–8.122)	0.210*	2.0 (1.54–6.28)	0.027*
	Secondary	6	41	1		1	1
	College and above						
Occupation	Governmental Employee	5	6	3.6 (1.67–6.43)	0.235*	2.58 (0.84–23.50)	0.079
	Private Employee	4	23	0.75 (0.25–5.24)	0.531	1.2 (1.24–6.14)	0.971
	Farmer	8	36	0.96 (0.75–5.24)	0.642	1.6 (1.65–7.13)	0.648
	Housewife	5	24	3.6 (1.68–8.79)	0.124*	2.1 (1.38–9.47)	0.098
	Student	11	53	5.45 (4.78–9.04)	0.008*	3.25 (0.91–22.95)	0.065
	Merchant	3	13	1	1	1	
Residence	Rural	29	97	2.4 (0.81–11.08)	0.614	1.933 (0.25–14.53)	0.52
	Urban	7	57	1		1	
Monthly income	< 50 USD	4	12	2.1 (2.93–8.32	0.969	0.84 (0.25–14.53	0.768
	50–100 USD	8	38	1.4 (1.62–7.48)	0.067	1.9 (1.96–8.63)	0.861
	100–200 USD	16	52	2 (3.03–7.31)	0.621	0.93 (0.31–2.73)	0.900
	> 200 USD	8	52	1		1	
Knowledge about Anti-Rabies Vaccination before	No	17	99	0.49 (0.86–2.72)	0.213*	1.34 (0.54–3.36)	0.527
	Yes	19	55	1	1	1	
Distance from the health center	> 20 km	19	91	0.6 (0.11–7.21)	0.122	2.49 (0.21–7.81)	0.042
	5–20 km	10	42	0.7 (0.31–57)	0.179	6.72 (1.62–29.23)	0.084
	< 5 km	7	21	1	1	1	1

*P* value < 0.05 is significant.

## Discussion

The study findings revealed significant gaps in PEP for rabies vaccination. Although the disease is preventable, it remains deadly among patients at Addis Alem General Hospital. The results indicate that increasing vaccination coverage in both humans and animals, improving accessibility for timely PEP, and raising awareness about disease prevention and treatment can help reduce the rabies burden in Ethiopia.

Over 53% of the study participants were young people, and half of them were students. Similar findings from studies conducted in Bangladesh and Tanzania also indicated that students accounted for a larger proportion of dog bite victims, possibly due to their travel to school and increased exposure to street dogs ^18,19^. Furthermore, about 66.3% of the participants lived in rural areas, which carry a higher risk of rabies. This may be due to the presence of unvaccinated, free-roaming animals or limited access to animal vaccination services. The findings are consistent with previous reports suggesting that rabies is strongly associated with exposure to domestic or stray animals and limited access to veterinary professionals in rural areas. This, in turn, contributes to poor vaccine access and limited knowledge about rabies and its treatment approaches in these communities ^18,19^.

Additionally, although the PEP regimen is very crucial, many people are not adhering to it. According to the results, while every individual received the first dose, nearly three percent did not receive the second dose, about eight people missed the third dose, approximately ten people missed the fourth dose, and nearly twelve people did not receive their fifth scheduled vaccination. The results showed a slight increase in missed doses as the vaccination schedule progressed from the first to the fifth dose. In total, 35 people missed one or more doses, while about 81.6% of participants adhered to the full vaccination schedule. This adherence rate is slightly higher than the rates reported in studies conducted in Chad (> 70%) ^20^ and China (62%) ^21^, but slightly lower than the findings from France (91%), which might be due to differences in vaccination accessibility ^22^. In Thailand, adherence to the vaccination schedule is higher, likely due to free vaccine access ^23^. The study conducted in Ethiopia also showed that only 57% of patients completed their treatment due to long distance from the health facilities, poor income, and accessibility issues ^24^.

Despite the Ethiopian government providing PEP vaccines for animals free of charge or at the lowest possible cost, transportation costs, accessibility, and affordability to human PEP remain challenges for many people ^25^. A study conducted in Ethiopia from 2015 to 2019 indicated that the average estimated cost needed for full rabies post exposure prophylaxis treatment is around 170 USD, which consequently leads for treatment discontinuation and missed doses due to the increasing cost of transportation and other related costs ^14^. A significant number of individuals failed to complete the five-dose vaccine schedule, highlighting the need to design strategies that minimize vaccine-related costs and improve public awareness through various platforms. Enhancing knowledge about the disease and the importance of completing the vaccination schedule is crucial to preventing complications and death. The investigation found that many people are not informed about rabies or how to prevent it ^26^. The study finding from Bangladesh also revealed similar results that even though the vaccine was given for free people showed 84% at dose 1 and only 16% at dose 5, due to fail to remember their schedule, deprived awareness, transportation difficulties, and family responsibilities ^27^.

Regarding knowledge, three out of four participants were aware of rabies, and nearly 40% of the people were previously familiar with anti-rabies vaccines. Most people received this information primarily from the radio (35.1%) and family members (28.4%), while a smaller number of participants obtained information through health education provided by healthcare workers. The study result showed comparable findings that only 14% of the participants are aware of the presence and location for PEP vaccination centers. Moreover, most of the respondents has poor awareness towards seeking PEP immediately after exposure and didn’t know that superficial wounds could spread the rabies virus infection ^28^. Many people do not understand the importance of receiving early post-exposure prophylaxis (PEP) after being bitten or scratched, nor are they aware of the risks associated with delayed vaccination or not being vaccinated at all. Among all exposed individuals, only 13.7% received the first dose of the vaccine on the same day as their exposure. About 42.6% sought vaccination at a health facility between 4 and 7 days after exposure. The delay in seeking vaccination increases the risk of poor treatment outcomes, highlighting the need to organize campaigns to improve public understanding of the benefits of early medical visits and the risks associated with delays ^29^.

Many young people showed better vaccination adherence compared to older individuals due to their higher likelihood of accessing information in today’s world, higher educational status, and closer connection to medical information. Adherence was comparatively higher among older adults than those under 20 years of age (AOR = 2.15, *p* = 0.023). The main reason might be that older individuals tend to focus more on their health and strictly follow their regimen. It is evident that education plays a prominent role in adherence, as many literate individuals follow their vaccination regimen more consistently than those with no formal education (AOR = 2.0, *p* = 0.027). Hence, education enhances understanding of the importance of adhering to the vaccination schedule.

Moreover, participants living closer to health facilities adhered better than those living more than 20 km away (AOR = 2.49, *p* = 0.042). This may be due to transportation costs, work-related challenges, and other factors, highlighting the need to improve accessibility and affordability while also increasing public awareness. People living near health facilities are more likely to receive the vaccine promptly after exposure. However, sex, occupation, and income level were not statistically significant.

Furthermore, the study findings underscore the need to improve adherence, along with enhancing awareness about rabies disease and its treatment options in various ways. Likewise, the importance of seeking early vaccination is highlighted as a crucial factor for improved PEP treatment outcomes after exposure. Due to the limited emphasis on preventive measures and insufficient knowledge about vaccination, poor adherence persists in many communities in developing countries ^25^. According to a study conducted in Bangladesh, many people visit health facilities for vaccination late due to lack of education, affordability issues, and reliance on traditional medicine for rabies treatment ^19^. Similarly, in Ethiopia, about 80% of the population relies on traditional medicine for primary healthcare, and a large number of people seek traditional treatment after a rabies bite ^15^. The study results showed comparable findings with studies conducted in Asian and African countries, where affordability, lack of awareness, and inadequate access continue to pose challenges in tackling rabies. Hence, adherence to vaccination schedules still requires various interventions, including improving access, reducing costs, and raising public awareness. Promoting vaccine uptake in Thailand through affordable vaccines and educational programs is an approach Ethiopia can learn from ^23^.

## Conclusion

Generally, the study findings showed that about 81.6% of people adhered well to their vaccination schedule at Addis Alem General Hospital. However, the results also highlight the need to address critical gaps to ensure the timely completion of post-exposure prophylaxis (PEP). Factors such as age, education level, and proximity to healthcare facilities influence adherence rates. Delays in seeking vaccination beyond three days remain a major challenge, as they increase the risk of rabies transmission and death. Similarly, poor public health awareness and reliance on informal information sources emphasize the need for enhanced health education. Ethiopia must work toward eliminating rabies in alignment with the WHO’s 2030 target, which requires strong community engagement, improved access to affordable vaccines, and strengthened early reporting and response systems.

## Limitation of the study

Since all participants were from a single site, the findings may not be generalizable to other settings. A multicenter study would enhance reliability and generalizability. Additionally, as data were collected at one point in time, causal relationships cannot be established.

## Data Availability

The datasets supporting the findings of this study are included within the manuscript.

## Abbreviations

AGH: Addis Alem general hospital
DALY: Disability-adjusted life years
HDCV: Human diploid cell culture rabies vaccine
KAP: Knowledge, attitudes and practices
MOH: Ministry of health
NTDs: Neglected tropical diseases
PEP: Post-exposure prophylaxis
SARE: Stepwise approach towards rabies elimination
WHO: World Health Organization
